# Infant Alveolar Macrophages Are Unable to Effectively Contain *Mycobacterium tuberculosis*

**DOI:** 10.3389/fimmu.2020.00486

**Published:** 2020-03-24

**Authors:** Anu Goenka, Ian E. Prise, Emma Connolly, Paulina Fernandez-Soto, David Morgan, Jennifer S. Cavet, John R. Grainger, Jaya Nichani, Peter D. Arkwright, Tracy Hussell

**Affiliations:** ^1^Lydia Becker Institute of Immunology and Inflammation, Division of Infection, Immunity, and Respiratory Medicine, University of Manchester, Manchester, United Kingdom; ^2^School of Cellular and Molecular Medicine, University of Bristol, Bristol, United Kingdom; ^3^Department of Paediatric Otolaryngology, Royal Manchester Children's Hospital, Manchester, United Kingdom

**Keywords:** macrophage, tuberculosis, infant, transcriptomics, chemokine, lung, lysosome

## Abstract

Infants are more likely to develop lethal disseminated forms of tuberculosis compared with older children and adults. The reasons for this are currently unknown. In this study we test the hypothesis that antimycobacterial function is impaired in infant alveolar macrophages (AMϕs) compared with those of adults. We develop a method of obtaining AMϕs from healthy infants using rigid bronchoscopy and incubate the AMϕs with live virulent *Mycobacterium tuberculosis* (Mtb). Infant AMϕs are less able to restrict Mtb replication compared with adult AMϕs, despite having similar phagocytic capacity and immunophenotype. RNA-Seq showed that infant AMϕs exhibit lower expression of genes involved in mycobactericidal activity and IFNγ-induction pathways. Infant AMϕs also exhibit lower expression of genes encoding mononuclear cell chemokines such as *CXCL9*. Our data indicates that failure of AMϕs to contain Mtb and recruit additional mononuclear cells to the site of infection helps to explain the more fulminant course of tuberculosis in early life.

## Introduction

Alveolar macrophages (AMϕs) are long-lived, tissue-resident phagocytes, originating from fetal monocytes colonizing the airways in the first days of life (1, 2). Their occasional replenishment in the alveolar airways by peripheral monocytes in the steady state is accelerated following inflammatory necrosis (3). AMϕs are trained by the lung microenvironment and their inflammatory responses are restrained to avoid inappropriate activation by harmless particulate matter and commensal microbes (2, 4). A number of pathogenic microorganisms exploit the AMϕ niche (5) including *Mycobacterium tuberculosis* (Mtb) (6).

Infants are particularly vulnerable to severe tuberculosis (TB), such as TB meningitis and miliary TB (7). Studies performed prior to the availability of effective antibiotics against Mtb demonstrated that bacillary dissemination occurs in approximately one third of infants, compared with only one in 20 older children and adults (7). Histology from these studies showed that infants with untreated TB exhibit dysfunctional granuloma formation and failure to control mycobacterial replication in the lung (8, 9). However, the facets of infant immunity responsible for these phenomena have not yet been elucidated.

An incomplete understanding of mycobacterial immunity has also hampered delivery of an effective vaccine against tuberculosis. Current candidate vaccines enhance antigen-specific T-cell immunity and IFNγ production (10), however Mtb disseminates before this develops (11). Animal studies have shown that Mtb-infected AMϕs are important in early bacillary containment, by initiating granuloma formation in the lung interstitium through the recruitment of other mononuclear cells (12–14). Human studies of primary AMϕs have demonstrated that these cells exhibit a unique transcriptional and functional response to Mtb, distinct from model systems of human macrophage immunity (15–17). No studies to date have examined Mtb control in primary AMϕs from human infants, predominantly due to difficulties in their sampling. We developed a method for obtaining AMϕs from infants without active clinical lung inflammation or infection and used this cellular source to show that infant AMϕs exhibit a multifaceted dysfunctional response to Mtb, including diminished ability to control Mtb replication. These data suggest that infant AMϕs are less able to contain the replication and spread of Mtb. This may relate to the lack of exposure to microenvironmental signals required to drive development of innate mycobacterial immunity in the infant (4, 18), and provides a potential approach to therapeutically train AMϕs in this vulnerable age group.

## Methods

### Human Subjects and Samples

The research protocol was approved by the National Health Service Research Ethics Committee (Reference 14/SW/0100 and 15/NW/0409). Written informed consent was obtained from adult participants and the legal guardians of infant participants in accordance with the Declaration of Helsinki. Bronchoalveolar lavage (BAL) with upto 20 mL 0.9% saline was performed on infants undergoing rigid bronchoscopy for suspected airway abnormalities at Royal Manchester Children's Hospital, using a ventilating Storz bronchoscope under sevoflurane inhalational anesthesia. In adults, BAL was performed with upto 50 mL 0.9% saline during flexible bronchoscopy for investigation of persistent unexplained cough or hemoptysis with normal thoracic imaging, as previously described (19). Exclusion criteria for participants undergoing BAL included any febrile illness within the last 14 days, receipt of antibiotics within previous 6 weeks, any evidence of immunodeficiency, immunosuppressive medication, previous close contact with an individual with TB, family history of TB, atopic disease, asthma, any chronic lung disease, any chronic inflammatory disease, or surgery involving general anesthetic within the last 4 weeks. Additional exclusion criteria for infant participants included preterm delivery (<37 weeks gestation), low birth weight (<2.5 kg) or congenital malformation.

Peripheral blood was obtained from participants undergoing BAL for IFNγ release assay (QuantiFERON-TB Gold Plus, QIAGEN) which was performed as per manufacturer's instructions. Lung resection samples were obtained from University Hospital of South Manchester through the Manchester Allergy, Respiratory and Thoracic Surgery Biobank.

### AMϕ Isolation and Culture

BAL fluid was transported to the laboratory on ice within 1 h of acquisition. Mucous was disrupted by the addition of a 4-fold volume of 0.1% dithiothreitol (Sigma-Aldrich) followed by gentle rocking for 15 min at room temperature. The suspension was filtered serially through sterile 150 and 50 μm filters (CellTrics) and diluted 2-fold with phosphate-buffered saline (PBS). Cells were either stained immediately for flow cytometry or prepared for culture by suspension in Roswell Park Memorial Institute (RPMI) 1640 Medium (Sigma-Aldrich) supplemented with 10% Fetal Calf Serum (FCS), 100 U/ml penicillin, 100 μg/ml streptomycin and 2 mM L-glutamine, and then seeded in 24-well tissue culture plates (Corning Inc.). Following 1h of incubation at 37°C/5% CO_2_, non-adherent cells were removed by vigorous washing with PBS, and adherent cells were cultured for downstream application.

To obtain sufficient cells with which to perform optimization experiments, AMϕs were harvested from healthy non-cancerous lung tissue (“*ex-vivo* BAL” AMϕs) obtained from patients undergoing surgery for suspected or confirmed cancer. These patients did not have a chronic inflammatory lung disease or severely impaired lung function (forced expiratory volume in 1 s/forced vital capacity ratio >70%). Lung tissue was perfused with PBS, followed by enrichment of mononuclear cells by Ficoll-Paque (GE Healthcare Biosciences) density gradient centrifugation according to the manufacturer's instructions. The mononuclear cells were either stained immediately for flow cytometry, or AMϕs were purified by adherence as described above.

### Bacteria

All handling of *M. tuberculosis* strains was performed under Biosafety Level 3 conditions with approval from the UK Health and Safety Executive. *M. tuberculosis* H37Rv (gift from Professor Brian Robertson, Imperial College London) was cultured at 37°C (without shaking) in Middlebrook 7H9 broth (BD Biosciences) containing 0.2% glycerol, 0.05% tween 80 and 10% OADC enrichment media (BD Biosciences). Mtb-LuxG13 (gift from Professor Brian Robertson, Imperial College London) was produced by transformation of *M. tuberculosis* H37Rv strain with a bacterial luciferase encoding vector (pMV306hsp + LuxAB + G13 + CDE, Kan^r^) allowing only live bacilli to produce both the substrate (*n*-decanal) and co-factor (FMNH2) required to generate light, as described previously (20). Except for addition of kanamycin 25 μg/mL (Sigma-Aldrich), Mtb-LuxG13 was cultured and harvested in the same way as *M. tuberculosis* H37Rv. Bacilli were harvested during midlog (OD_600nm_ 0.4–0.7) growth and washed with PBS, before suspension in cell culture medium at the desired concentration following declumping by serial passage eight times through a blunt 26G needle (SAI Infusion Technologies).

### Macrophage Infection Assays

Mtb-LuxG13 was added to 2 × 10^5^ freshly purified AMϕs at the appropriate multiplicity of infection (MOI) in antibiotic-free RPMI 1640 media supplemented with 10% FCS and 2 mM L-glutamine, and was incubated at 37°C/5% CO_2_ in sterile white transparent-bottom 24-well luminometry tissue culture plates (Berthold Technologies). Following incubation for 2 h, AMϕs were washed three times with phosphate-buffered saline to remove extracellular bacteria, and fresh culture medium was added. CFU counting of: (i) the inoculum confirmed MOI; (ii) macrophage lyste confirmed the proportion of bacilli phagocytosed, whenever sufficient AMϕs were available, as previously described (20). Serial measurements of autoluminescence were performed by a LUMIstar Omega (BMG LABTECH) plate luminometer during a 48–72 h incubation period, because beyond this there was obvious cell death of AMϕs as indicated by their non-adherence and trypan blue staining. Autoluminescence was cumulatively measured as Relative Light Units (RLU) over 10 s via the bottom reading optic, and CFU assay correlations were performed as previously described (20). After 24 h of incubation, 50 μL of culture supernatant was removed, sterilized and stored at −80°C for cytokine bead array.

### Flow Cytometry

Cells were incubated at 4°C for 20 min with Live/Dead stain (Zombie UV Fixable Viability Kit, BioLegend) and mouse serum (Sigma-Aldrich). After washing with PBS, cells were incubated at 4°C for 20 min with the appropriate antibody cocktail (Panel 1: CD40 BV785, TLR2 AF700, CD200R AF647, CD119 APC-Vio770, CD3 FITC, CD19 FITC, CD56 FITC, CD66b FITC, CD235a FITC, CD64 PE-Cy7; or Panel 2: Sirpα APC, HLA-DR AF700, CD206 APC-Cy7, CD3 FITC, CD19 FITC, CD56 FITC, CD66b FITC, CD235a FITC, CD64 PE-Cy7), before washing and fixation with 3.7% paraformaldehyde at room temperature. Data were acquired on a BD Fortessa cytometer (BD Biosciences). In all experiments, single stain controls were prepared using compensation beads (OneComp eBeads, Fisher Scientific) and were used to standardize voltage settings. At least 50,000 cells were acquired from macrophage samples. Samples were analyzed after compensation was set using FlowJo (Version 10.3, Tree Star), and gating to determine percentage positive expression was determined using the fluorescence-minus-one principle.

### Cytokine Bead Array

Culture supernatant was sterilized by 0.22 μm cellulose acetate membrane centrifuge tube filtration (Corning Inc.) before removal from Biosafety Level 3 conditions and storage at −80°C. After thawing, soluble inflammatory mediator production was quantified by multiplex cytokine bead array as per the manufacturer's instructions (Soluble Protein Human Flex Set, BD Biosciences). Briefly, supernatants were incubated with cytokine detection beads alongside a phycoerythrin-conjugated detection protein. Recombinant cytokine was analyzed to produce a standard curve to fit the measurements of supernatant samples, with a lower limit of detection was 20 pg/mL for all cytokines. To allow sample measurement to fall within upper limit of detection for TNFα, IL6, IL8, and CCL4, it was necessary to pre-dilute supernatants 50-fold with assay buffer. Samples were acquired on a BD FACSVerse system, and data analyzed using FCAP Array (Version 3.0, Soft Flow Inc.).

### RNA Isolation

Lysate from AMϕs was stored at −80°C following cell disruption with buffer RLT (QIAGEN) containing 1% β-mercaptoethanol (Sigma-Aldrich). RNA was isolated from lysates using RNeasy Micro Kit (QIAGEN) according to the manufacturer's instructions. RNA was quantified using a Qubit 2.0 Fluorimeter (Thermo Fisher Scientific). RNA samples were assessed using a 2200 TapeStation (Agilent Technologies) and deemed of acceptable quality if they had an RNA Integrity Number (RIN) of greater than 8.0.

### RNA-Seq

The TruSeq® Stranded mRNA assay (Illumina Inc.) was used to generate libraries according to the manufacturer's protocol. The loaded flow-cell was then paired-end sequenced (76 + 76 cycles, plus indices) on an Illumina HiSeq4000 instrument. Finally, the output data was demultiplexed allowing one mismatch and converted to fastq format by bcl2fastq software (Version 2.17.1.14, Illumina Inc.). The quality of the unmapped paired-end sequences was assessed by FastQC (Version 0.11.7, Babraham Institute). Trimmomatic (Version 0.36) was then used to trim sequence adaptors and low-quality reads. Reads were mapped against the reference human genome (hg38) and counts per gene were calculated using annotation from GENCODE 27 using STAR (Version 2.5.3). The minimum proportion of reads that were uniquely mapped and counted into annotated genes was 80%.

Normalization of uniquely mapped reads was calculated with DESeq2 (Version 1.16.1) using the median of ratios method that accounts for RNA composition and sequencing depth, after which the software performed principal component analysis (PCA) and calculated differential expression using the multiple comparison correction of Benjamini-Hochberg on differentially expressed (DE) genes in which a False Discovery Rate (FDR) <0.05 was considered significant. Gene ontology (GO) enrichment analysis (AmiGO2, Version 2.5) and identification of upstream regulators of gene expression (Ingenuity Pathway Analysis, QIAGEN, Version 44691306) were performed on DE genes with a FDR <0.05 (Fisher's Exact with FDR multiple test correction) and a real fold change >2. Gene set enrichment analysis (GSEA) was performed as described previously (21).

### Statistical Analysis

Statistical analysis of *in vitro* functional data was undertaken using Prism (Version 7.0, GraphPad Software). Parametric distribution of the data was confirmed by the Shapiro-Wilk normality test. The significance levels were set at *p* ≤ 0.05 and FDR ≤ 0.05.

## Results

### Recovery of Infant Alveolar Macrophages From Non-inflamed Infant Lungs

To obtain AMϕs from infants without active clinical inflammation we performed bronchoalveolar lavage (BAL) during rigid bronchoscopy for the investigation of suspected airway abnormality. Since BAL is usually performed by flexible bronchoscopy for the investigation of infection/inflammation (22), this required the development of a method of instilling/recovering saline from the lower respiratory tract (Supplementary Video 1). Using this technique, we obtained BAL from 20 infants. We also performed BAL on 20 adults by flexible bronchoscopy (Table 1).

**Table 1:** Baseline characteristics and macrophage yield of participants.

	**Infants** **(*n* = 20)**	**Adults** **(*n* = 20)**
**Age**, median (range)	11 months (6-23)	59 years (36-78)
**Gender**		
Male	9 (45%)	11 (55%)
Female	11 (55%)	9 (45%)
**Ethnicity**		
White European	18 (90%)	19 (95%)
Asian	2 (10%)	1 (5%)
**Clinical indication** (*n*, %)	Stridor (13, 65%) Cyanotic episodes (4, 20%) Recurrent croup (2, 10%) Hoarse cry (1, 5%)	Persistent cough (11, 55%) Haemoptysis (9, 45%)
**Diagnosis** (*n*, %)	Normal (11, 55%) Laryngomalacia (6, 30%) Laryngeal web (1, 5%) Tracheomalacia (2, 10%)	Normal (20, 100%)
**BAL fluid instilled** (mL), median (range)	15 (5-20)	50 (40-100)
**BAL fluid retrieval** (%), median (range)	20 (5-40)	40 (20-66)
**AMϕ** **yield** (cells × 10^5^), median (range)	2.9 (1.8–18)	2.35 (1.2–13)

None of the infants had received BCG vaccination, while all adults had received BCG vaccination during childhood/adolescence. None of the participants had a contact history with an individual with Mtb or a family history of Mtb. The QuantiFERON-TB Gold Plus assay that analyses IFNγ release to Mtb-specific antigens was negative (IFNγ < 0.35 IU/L) in all participants. None of the infants had a history of chronic illness, and chronic conditions in adults included hypertension in 8/20 (40%), hypercholesterolemia in 6/20 (20%), ischemic heart disease in 4/20 (20%), type 2 diabetes mellitus in 4/20 (20%) and breast cancer in remission in 2/20 (10%). In adults, 8/20 (40%) had never smoked, 6/20 (30%) identified as an ex-smoker and 6/20 (30%) were current smokers. Procedures were well tolerated by all participants. Macrophage viability was >98% in all samples as assessed by trypan blue exclusion. Due to limited cell numbers, each sample was only able to contribute to one experiment (Supplementary Table 1).

### Infant and Adult AMϕs Express Similar Levels of M1/M2 Activation Markers

**Figure 1:**
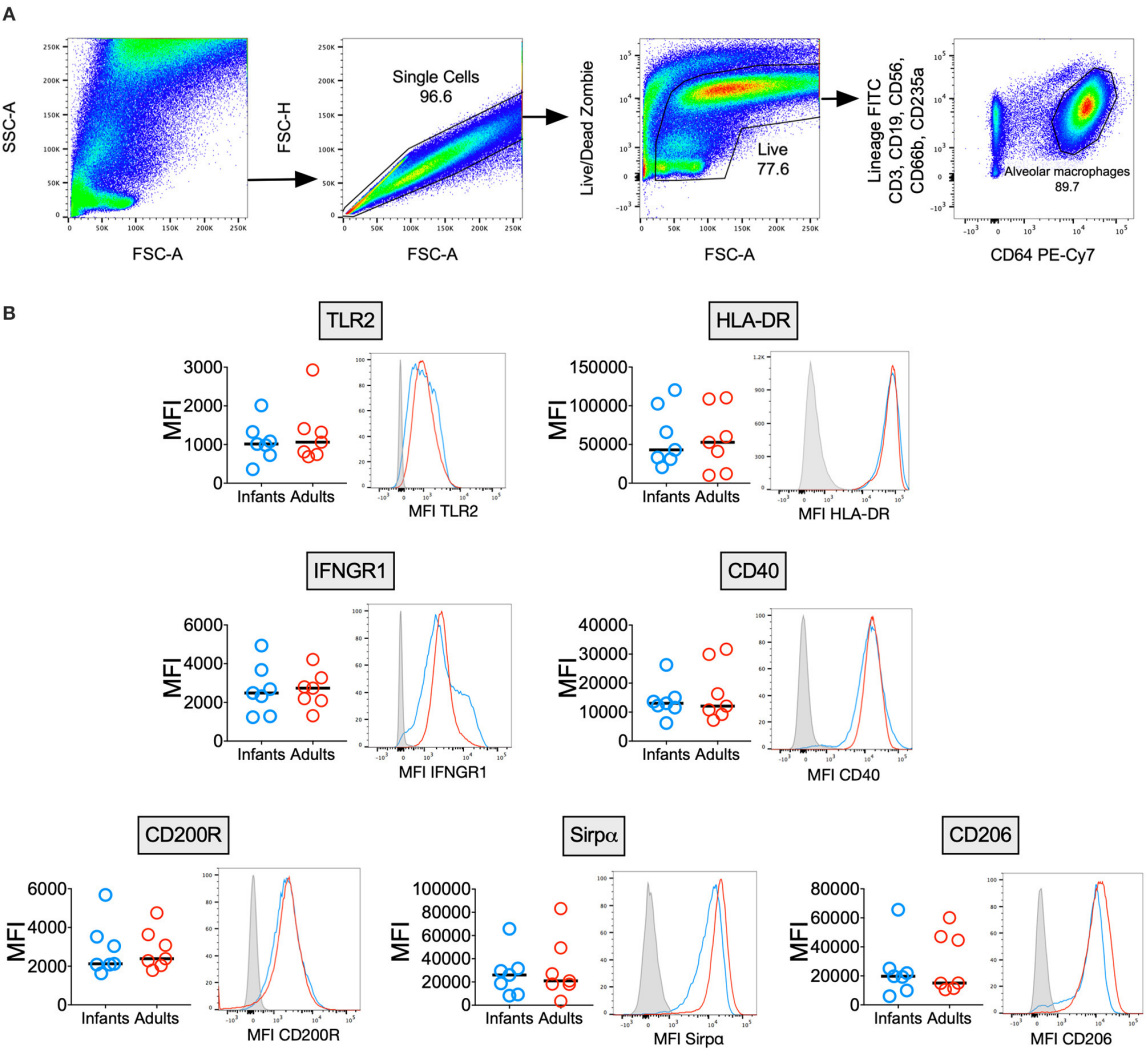
Infant and adult AMϕs have a similar immunophenotype. **(A)** Representative flow cytometry plots showing gating strategy to differentiate AMϕs based on their high autofluorescence (in the 488 530/30 FITC channel) and high CD64 expression, from lineage positive cells (CD3+ T cells, CD19+ B cells, CD56+ NK cells, CD66b+ granulocytes, and CD235a+ erythrocytes) and monocytes (low autofluorescence in 488 530/30 FITC channel, lineage negative and CD64+). **(B)** Flow cytometry of AMϕs from seven infant BAL samples (blue representative histogram) compared with seven adults (red representative histogram), with fluorescence minus one (gray representative histogram). Line represents median. MFI, mean fluorescence intensity.

AMϕs are known to express a unique combination of phenotypic markers due to the influence of the airway microenvironment (2), but few reports have described the immunophenotype of human infant AMϕs (23). We measured surface marker expression of AMϕs from seven infants and seven adults (Figure 1). There was no significant difference in the expression of markers reflecting classical M1-activation relevant to antimycobacterial function: TLR2 (mycobacterial recognition), HLA-DR (antigen presentation) (24), CD40 (co-stimulation) (25) and IFNGR1 (activation) (26). Similarly, there was no significant difference in markers indicating alternative M2-activation: CD200R, Sirpα and CD206 (2).

### Impaired Mtb Control and Altered Chemokine Production by Infant AMϕs

To compare the ability of infant vs. adult AMϕs to restrict bacillary replication, we optimized a reporter assay that uses the autoluminescent strain Mtb-LuxG13 for use with primary human AMϕs (Supplementary Figure 1). Mtb-LuxG13 is produced by transformation of *M. tuberculosis* H37Rv with bacterial luciferase, which confers only live bacilli with the ability to generate light (20). Bacterial autoluminescence correlated with CFU in liquid broth culture and following infection of AMϕs (Supplementary Figures 1A–D). Infection of AMϕs with a MOI 10:1 was associated with a higher autoluminescence (reflective of bacillary load) compared with MOI 5:1, despite a similar proportion of phagocytosed bacilli and replication rate, which was estimated from the fold-change in autoluminescence (Supplementary Figures 1E,F). Consistent with its putative biological function in mouse macrophages (5), treatment with exogenous IFNγ was associated with reduced bacillary replication in AMϕs infected with Mtb-LuxG13, compared with untreated cells (Supplementary Figure 1G). Therefore, our assay yielded accurate and reliable measurements of mycobacterial phagocytosis and capacity to restrict mycobacterial replication by human AMϕs.

**Figure 2:**
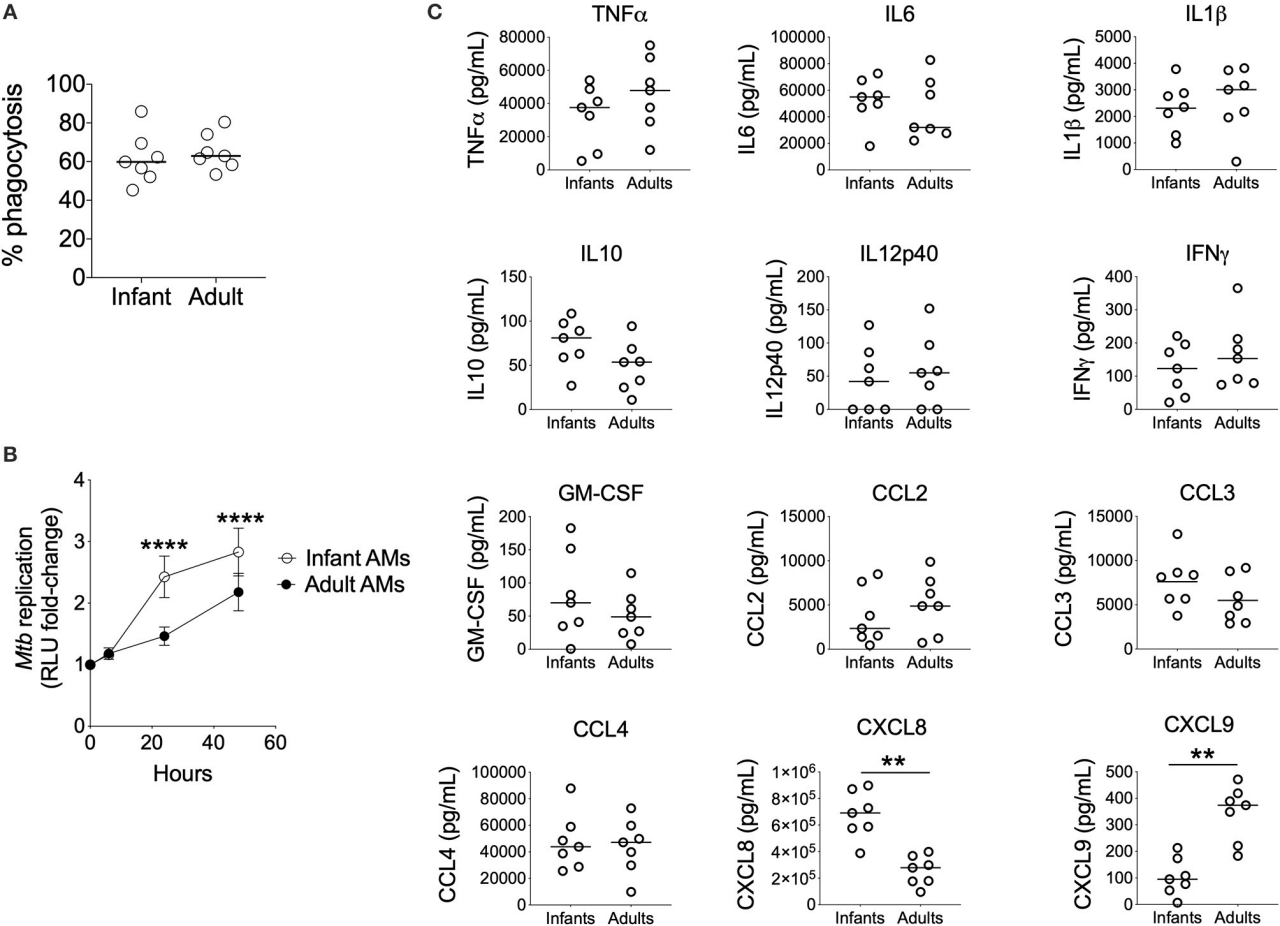
Impaired control of Mtb by Infant AMϕs. **(A)** Phagocytosis of Mtb-LuxG13 by AMϕs from seven infants and seven adults after 2 h of incubation at MOI 5:1. Proportion of phagocytosed bacteria assessed by measurement of autoluminescence after removal of extracellular bacilli with PBS washes, as a percentage of autoluminescence before PBS washes. Each data point represents a single observation from a single participant. Bar denotes median; **(B)** Ability of AMϕs to restrict replication of Mtb-LuxG13. Data points represent mean (bar = SD) of AMϕs from seven infants (empty circles) compared with seven adults (filled circles), and line shows fold-change in autoluminescence calculated from serial measurements relative to zero time point, following 2 h of incubation with Mtb-LuxG13 at MOI 5:1. Significance determined by 2-way ANOVA and denoted by ****(*p* < 0.0001); **(C)** Soluble inflammatory mediator production in culture supernatants by infant vs. adult AMϕs at 24 h post infection with Mtb-LuxG13. Significance determined by Mann-Whitney test. Statistically significant results denoted by **(*q* < 0.01) and after correction for multiple comparisons by Benjamini-Hochberg method.

Using this assay, we found no significant differences in the phagocytosis of Mtb-LuxG13 by AMϕs from seven infant vs. seven adult participants (Figure 2A). However, the fold-change in mycobacterial autoluminescence (reflective of mycobacterial replication) was significantly higher in infant AMϕs at 24 h (*p* < 0.0001) and 48 h (*p* < 0.0001) post-infection, compared with adult equivalents (Figure 2B). This difference was associated with an extended lag prior to mycobacterial replication in adult AMϕs in the first 24 h post-infection, with an estimated mean doubling time of 58 h in adult AMϕs, compared with 18 h in infant AMϕs. In addition, infant AMϕs produced significantly more CXCL8 (*q* = 0.007) in culture supernatants at 24 h post-infection, but significantly less CXCL9 (*q* = 0.007) compared with adult AMϕs (Figure 2C). There was no significant difference in the production of TNFα, IL6, IL1β, IL10, IL12p40, IFNγ, GM-CSF, CCL2, CCL3, or CCL4. We conclude that infant AMϕs were less capable of restricting Mtb replication and exhibit altered chemokine production compared with adult counterparts.

### Transcriptional Disparity Between Mtb-Stimulated Infant vs. Adult AMϕs

To define why infant AMϕs were less capable of controlling Mtb replication, we performed two RNA-Seq experiments to compare gene expression of: (i) AMϕs from four infants and four adults following *in vitro* infection with *M. tuberculosis* H37Rv for 24 h; and (ii) freshly isolated AMϕs following 1 h adherence four infants and four adults.

**Figure 3:**
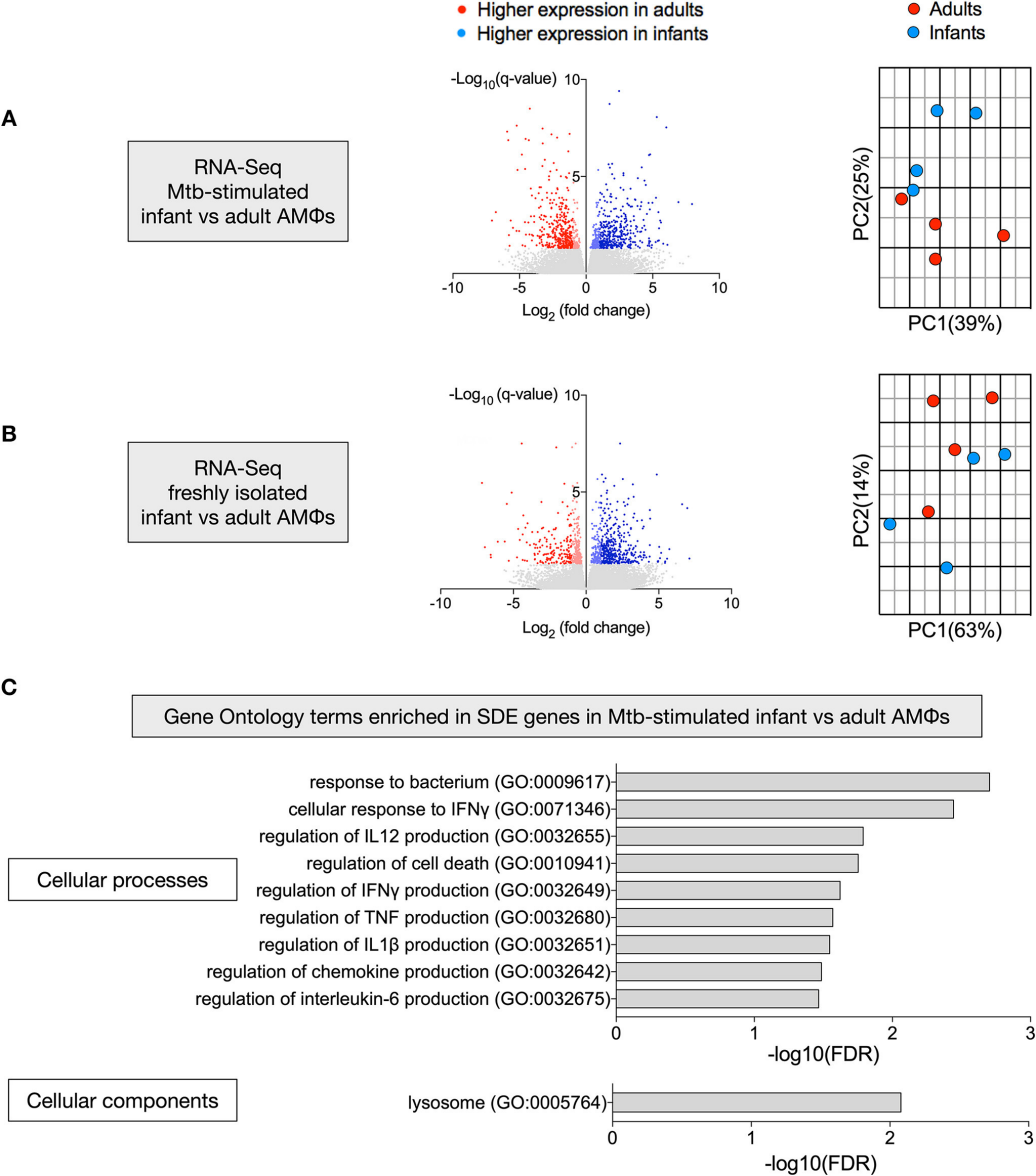
Transcriptional disparity between Mtb-stimulated infant vs. adult AMϕs. RNA-Seq of infant vs. adult AMϕs following **(A)** infection with Mtb H37Rv at MOI 5:1 for 24 h; and **(B)** adherence of freshly isolated cells for 1 h. For volcano plots (left), blue dots represent genes that were significantly (FDR < 0.05) differentially expressed (SDE) more highly in infants [log2(fold change>1 infant expression/adult expression)], and red dots represent genes that were SDE more highly in adults [log2(fold change < -1 infant expression/adult expression)]. For Principal Component Analyses (right), blue dots represent each individual infant participant and red dots represent each individual adult participant. **(C)** Gene ontology (GO) enrichment analysis of SDE genes in Mtb-stimulated infant vs. adult AMϕs showing significantly enriched GO terms for cellular processes (top) and cellular components (bottom).

There were 768 significantly differentially expressed (SDE) genes between infants and adults in the Mtb-stimulated samples (Figure 3A), compared with 591 SDE genes in the freshly isolated samples (Figure 3B). Consistent with this, the second principal component (25% variance) separated Mtb-stimulated samples from infant and adult participants (Figure 3A), but there was no infant vs. adult separation in a separate PCA of the freshly isolated samples (Figure 3B). Evaluation of SDE genes in Mtb-stimulated infant vs. adult AMϕs using gene ontology (GO) enrichment analysis revealed over-representation of genes involved in cellular processes and components relevant to mycobacterial immunity (Figure 3C).

### Polarized Gene Expression Affecting Specific Functional Pathways in Mtb-Infected AMϕs

**Figure 4:**
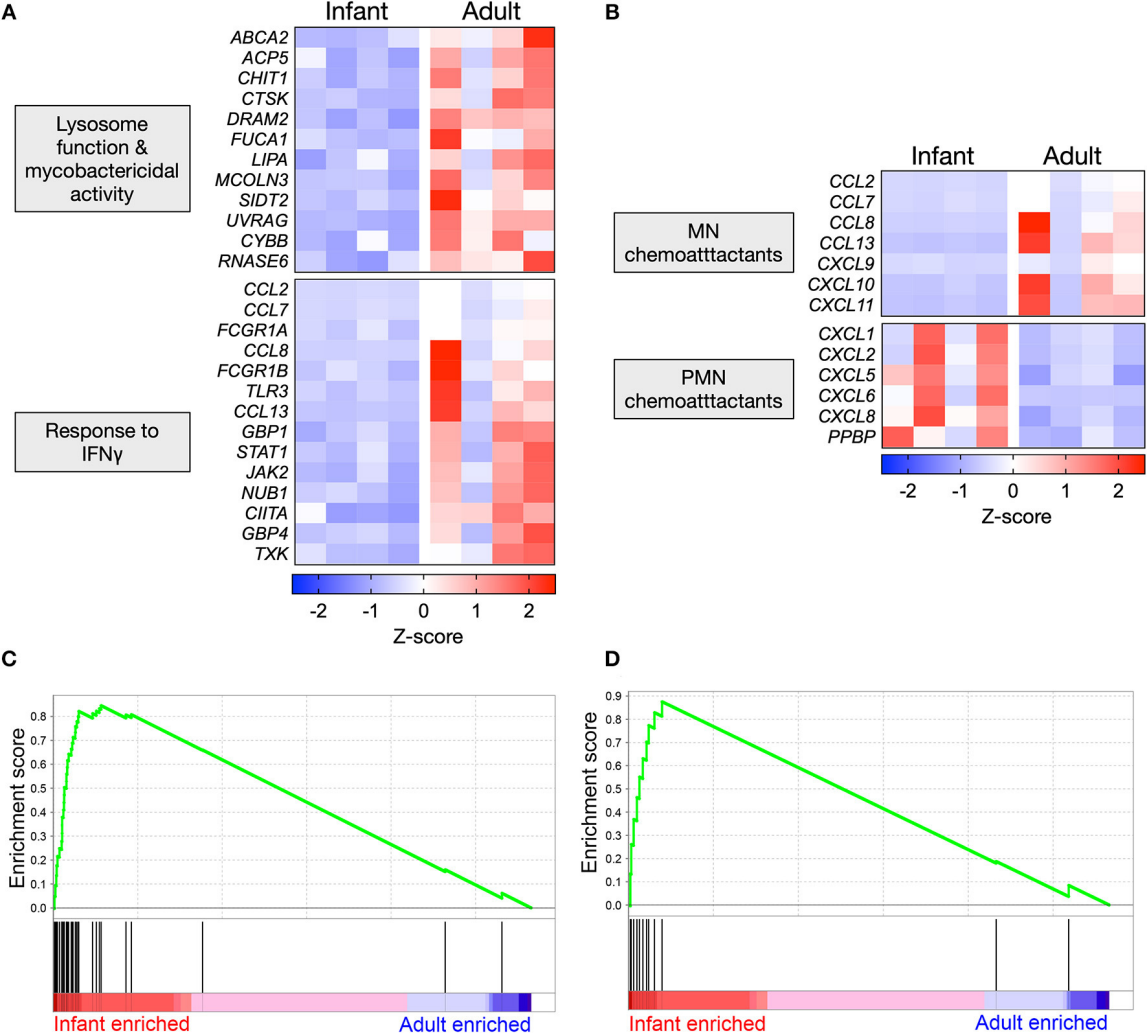
Polarized gene expression affecting specific functional pathways in Mtb-infected AMϕs. **(A)** Expression profile of SDE genes in Mtb-stimulated infant vs. adult AMϕs involved in lysosome function (GO: 0000323), response to bacterium (GO:0009617) and cellular response to IFNγ (GO: 0071346); and **(B)** Expression profile of all SDE genes encoding chemokines in Mtb-stimulated infant vs. adult AMϕs. Scale intensity represents Z-score. **(C)** Enrichment of 36 genes in the transcriptome of Mtb-stimulated infant/adult AMϕs previously shown to be upregulated in AMϕ in response to H37Rv Mtb relative to the avirulent H37Ra Mtb strain in (27); and **(D)** Enrichment of 12 genes in the transcriptome of Mtb-stimulated infant/adult AMϕs previously shown to be upregulated in Mtb-stimulated monocyte-derived macrophages from patients who had previously recovered from TB meningitis and pulmonary TB, relative to equivalents from patients with latent TB (28). GSEA plots showing all genes from Mtb-stimulated dataset ranked horizontally from highest differential expression in infants (red) to highest differential expression in adults (blue). The enrichment profile (green line) shows the degree of overrepresentation of previously published sets of genes (vertical black lines). Analysis performed using GSEA software (Version 6.3, Broad Institute).

Mtb-stimulated infant AMϕs exhibited lower expression of genes that promote lysosomal maturation and mycobactericidal activity in comparison with adult equivalents (Figure 4A). Consistent with the established role of IFNγ in initiating a transcriptional program that results in mycobacterial killing, we found that Mtb-stimulated infant AMϕs also exhibited lower expression of genes involved the cellular response to IFNγ, including *JAK2* and *STAT1* (Figure 4A). Congruent with this, IPA Upstream Regulator analysis predicted that inhibition of IFNγ in infant AMϕs was the most statistically significant (overlap *p*-value = 1.03E-51, activation z-score = −2.8) upstream factor responsible for the pattern of Mtb-stimulated SDE genes that we had observed.

We also examined all SDE genes encoding chemokines and found that Mtb-stimulated infant AMϕs exhibited a lower expression of all genes encoding mononuclear chemoattractants compared with adult equivalents (Figure 4B). In contrast, Mtb-stimulated infant AMϕs also displayed higher expression of all genes encoding neutrophil chemoattractants compared with adult equivalents (Figure 4B). Of the chemokines measured in culture supernatants, a similar trend was observed for CXCL8 which was higher in infants, and CXCL9 which was lower in infants (Figure 2C and Supplementary Figure 2).

Overall the infant vs. adult gene expression differences in Mtb-stimulated AMϕs (lysosome function, mycobactericidal activity, response to IFNγ and chemokine expression) appeared relatively specific, because there was no clear infant vs. adult pattern of gene expression among enriched GO terms for other important antimycobacterial functions such as the regulation of innate cytokine production (IFNγ, IL12, TNF, IL6, and IL1β) and cell death (Supplementary Figure 3A). There was also no clear infant vs. adult pattern of expression of genes encoding Mtb phagocytosis receptors (Supplementary Figure 3B) (29–31).

We also tested whether the gene expression exhibited by Mtb-infected infant or adult AMϕs may be concordant with previously published transcriptomic studies of Mtb-stimulated macrophages (27, 28), using Gene Set Enrichment Analysis (GSEA). We found significant overrepresentation of genes that have previously been associated with infection of AMϕs with Mtb H37Rv vs. the avirulent strain H37Ra (*p* = 0.03) (Figure 4C, Supplementary Table 2) as well as genes associated with severe clinical TB (*p* = 0.048) (Figure 4D, Supplementary Table 3) in our transcriptomic data of Mtb-infected infant AMϕs, relative to adult equivalents.

We then examined RNA-Seq data from the freshly isolated samples to ascertain whether differential infant vs. adult gene expression in the steady state condition might predict the differences that we had observed in the Mtb-stimulated samples. Of the 13 SDE genes encoding chemokines in the Mtb-stimulated samples, two neutrophil chemoattractants (*CXCL1* and *CXCL2*) were significantly more highly expressed in freshly isolated infant AMϕs compared with adult equivalents. Of the 26 Mtb-stimulated SDE genes involved in IFNγ signaling, lysosome function and mycobactericidal activity, none were differentially expressed in freshly isolated infant vs. adult AMϕs. Therefore, the infant vs. adult differences in gene expression observed in Mtb-stimulated AMϕs were not observed in the steady state condition.

We also undertook GO enrichment analysis of the SDE genes from freshly isolated samples to identify other areas of cellular functioning that may differ between infant and adult AMϕs in steady state conditions. This unexpectedly revealed significant enrichment (FDR 4.78E-07) of genes involved in regulation of cell cycle, in which freshly isolated infant AMϕs exhibited higher expression of genes involved in both the positive and negative regulation of cell cycle, compared with adult equivalents (Supplementary Figure 4).

## Discussion

We provide the first clinically relevant comparison of infant vs. adult human AMϕs. Following Mtb infection, infant AMϕs were less able to restrict mycobacterial replication, despite similar degrees of bacterial phagocytosis. They also exhibited reduced expression of genes involved in lysosome function, mycobactericidal activity and response to IFNγ. Furthermore, Mtb-stimulated infant AMϕs also exhibited lower expression of chemokines that recruit mononuclear cells and exhibited higher expression of chemokines that recruit neutrophils. Our results are consistent with historical autopsy studies of untreated infants with TB, in whom granulomas are characterized by bacillary outgrowth, fewer mononuclear cells and increased neutrophils (8, 9). The clinical relevance of our data is further illustrated by enrichment of a previously described set of genes associated with disseminated TB in the transcriptome of our Mtb-stimulated infant AMϕs (28).

The key role of IFNγ in mycobacterial immunity has been defined in part by monogenic defects in the “IFNγ/IL12 circuit” that cause susceptibility to avirulent mycobacterial infection, collectively termed Mendelian Susceptibility to Mycobacterial Disease (32). Functionally, ligation of the IFNγ receptor of Mtb-infected murine macrophages results in antimycobacterial effector action such as ROS production, phagolysosome maturation, autophagy and cytokine/chemokine production (5). However, few previous studies have tested the effect of exogenous IFNγ on Mtb replication in human primary AMϕs (15, 33). Using an autoluminescent reporter assay, we demonstrate a reduced rate of Mtb replication in IFNγ-treated AMϕs from adult participants. Importantly, our data also show that infant AMϕs may be intrinsically less responsive to IFNγ compared with adult equivalents, and that IFNγ is a master regulator of the transcriptional differences observed. The unresponsiveness of infant AMϕs to IFNγ might be caused by their lack of exposure to viral infections, which have been shown to drive training and “innate macrophage memory” through CD8+ T cell-mediated priming of AMϕs by IFNγ (4). Infant AMϕs also exhibited lower expression of the IFNγ-stimulated gene (ISG) *CYBB*, which encodes a membrane-bound subunit of the nicotinamide adenine dinucleotide phosphate (NADPH) oxidase system responsible for ROS production, mutations of which are associated with susceptibility to mycobacterial infection (34). In addition, infant AMϕs exhibited lower expression of genes involved in antimycobacterial phagosome maturation (*DRAM2* and *UVRAG*) that are not known to be ISGs (35). Alongside this, infant AMϕs exhibited lower expression of genes involved in lysosome functioning (*ACP5* and *FUCA1*), including cholesterol breakdown (*LIPA*) and efflux (*ABCA2*) (36–38). Mtb preferentially utilizes host cholesterol as a fuel source and reduced cholesterol breakdown may lead to intracellular persistence of bacteria (39). Furthermore, lysosomal dysfunction is associated with impaired macrophage migration (40), which may lead to poorer containment of Mtb (14).

As the first cells to be infected by Mtb, AMϕs are key producers of chemokines (5). Recruitment of mononuclear cells is non-redundant in mycobacterial immunity and granuloma formation (41). For example, *Ccr2*^−^^/–^ and *Cxcr3*^−^^/–^ mice exhibit decreased monocyte and lymphocyte recruitment and dysfunctional granuloma formation (42, 43). In particular, chemokines that signal through CXCR3 (CXCL9, CXCL10, and CXCL11) recruit protective IFNγ-producing T helper type 1 cells (42). We observed that infant AMϕs displayed lower expression of all seven SDE genes encoding mononuclear chemokines that signal through three receptors: CCR1 (*CCL7, CCL8, CCL13*), CCR2 (*CCL2, CCL7, CCL8, CCL13*), and CXCR3 (*CXCL9, CXCL10, CXCL11*) (41). We also show that infant AMϕs display higher expression of genes encoding chemokines that attract neutrophils through CXCR1 and CXCR2 (*CXCL1, CXCL2, CXCL5, CXCL6, PPBP, CXCL8*). Neutrophil accumulation may be detrimental, as demonstrated by observations of Mtb-infected necrotic neutrophils promoting mycobacterial outgrowth, and improved survival of Mtb-infected mice if neutrophil infiltration is inhibited (44, 45). Vulnerability to severe TB and dysfunctional granuloma production in infants may therefore partly occur through disordered chemokine production by their AMϕs.

We found that unstimulated infant AMϕs exhibited higher expression of genes involved in both the positive and negative regulation of cell cycle compared with adult equivalents. Early childhood is a period of rapid structural change in the lung, including exponential increase in the number of alveoli (46). Investigators estimate that each alveolus contains up to five AMϕs (47), and so the dominance of cell cycle associated genes may reflect macrophage expansion to fill the developing niche.

The main limitation of this study is the small number of participants. A greater number of participants may help with understanding the variability within the infant and adult participant groups. Adult participants should ideally be relatively young, as macrophage function may be impaired in older adults. Furthermore, we cannot rule out if macrophage function was confounded in some participants by exposure to cigarette smoke, inhalational anesthesia, or gastro-esophageal reflux which commonly co-exists with laryngomalacia (48–50). Another potential source of confounding in our study was that adults, but not infants, had received BCG vaccination, which has been shown to educate hematopoietic stem cells to produce trained monocytes and macrophages (51). Despite these limitations we were able to elicit statistically significant functional and transcriptomic differences between infant and adult AMϕs. Now that feasibility of sampling is demonstrated, our data should prompt future studies that comprehensively compare infant and adult lung microenvironments, in particular factors known to affect early control of Mtb infection such as interstitial macrophages, dendritic cells, alveolar epithelial cells, respiratory microbiota and soluble factors/opsonins (14, 52, 53). Ideally, these studies would also define further the extent of infant AMϕ dysfunction in mycobacterial immunity, including areas that we did not specifically assess functionally (e.g., cell death, response to IFNγ, phagolysosomal maturation, autophagy, eicosanoid production) as well as mechanistic studies to better understand the relevance of infant AMϕ dysfunction described in our data (e.g., chemokine production) (5). This work should also assess the expression of bacterial virulence factors in Mtb-infected infant AMϕ compared with adult equivalents, given our finding that a previously determined transcriptomic signature of mycobacterial virulence was significantly enriched in our Mtb-stimulated infant AMϕ transcriptomic data (27). Non-human primate models could explore whether the observed pattern of AMϕ dysfunction in the human infant results in delayed recruitment of mononuclear cells, impaired Th1 immunity and granuloma formation, and increased haematogenous spread of bacilli (54).

Novel and fundamental insights into mycobacterial immunity are required to overcome the current impasse in TB vaccination and therapeutics. Taken together, our results provide the first evidence that age-dependent differences in AMϕ function may contribute to clinical vulnerability to TB. Improved understanding of the age-dependent microenvironmental factors that may drive trained immunity of AMϕs may inform the design of novel therapeutics with broad clinical applications against infectious and allergic disease.

## Data Availability Statement

The RNA-Seq data for this study can be found in the ArrayExpress database (EMBL-EBI) under accession number E-MTAB-7679.

## Ethics Statement

The studies involving human participants were reviewed and approved by National Health Service Research Ethics Committee (Reference 14/SW/0100 and 15/NW/0409). Written informed consent to participate in this study was provided by the participants' legal guardian/next of kin.

## Author Contributions

AG, PA, and TH conceived the idea for the study and designed the experiments. JN assisted setting up the infant bronchoalveolar lavage methodology and obtained clinical samples from infant participants. PF-S and JC assisted with initiating the macrophage infection assays. AG collected the samples, performed the experiments, and received assistance from DM and EC. AG analyzed the RNA-Seq experiment with assistance from IP. AG wrote the first draft of the manuscript. All authors contributed to critical review of the manuscript and approved the manuscript for submission.

### Conflict of Interest

The authors declare that the research was conducted in the absence of any commercial or financial relationships that could be construed as a potential conflict of interest.
