# Supplementary material for: Infant Alveolar Macrophages Are Unable to Effectively Contain Mycobacterium tuberculosis
Source: Front Immunol. 2020 Mar 24;11:486. doi: 10.3389/fimmu.2020.00486 (PMC7107672; doi:10.3389/fimmu.2020.00486)
Supplement: Supplementary file 1 [file Data_Sheet_1.pdf]

Supplementary Material

Supplementary Table 1. Bronchoscopy Diagnosis of Infant Participants and Smoking Status of Adult Participants

	Bronchoscopy Diagnosis of Infants (n)				Smoking Status of Adults (n)		
	Normal	Laryngo-malacia	Laryngeal web	Tracheo-malacia	Never	Ex	Current
**Figure 1** (n=7)	5	2	0	0	3	2	2
**Figure 2** (n=7)	4	2	0	1	2	3	2
**Figure 3** (n=6*)	4	0	1	1	3	1	2

* Bronchoalveolar lavage from 2 infants and 2 adult participants yielded sufficient AMϕs for use in both the freshly isolated and Mtb-stimulated RNA-Seq experiments.

Supplementary Table 2. List of transcripts included in GSEA of Mtb-stimulated infant/adult AMϕ that have previously shown to be upregulated in AMϕ in response to H37Rv Mtb relative to the avirulent H37Ra Mtb strain (1)

GADD45A
AKAP2
CCL3
CD44
*CSF2**
*CXCL1**
*CXCL2**
DUSP5
EHD1
*EREG**
*G0S2**
HIVEP2
HSPA1A
HSPA1B
IER3
*IL23A**
IL6
IVNS1ABP
LAMP3
*LIMK2**
OPTN
PALM2
PHLDA2
*PLA2G4A**
*PTGS2**
RGS1
SEC24A
SERPINB9
STK17A
*TFPI2**
TM4SF1
TNF
TNFAIP2
TNIP1
*TRAF1**
XBP1

*Genes that were SDE between Mtb-stimulated infant vs adult AMϕs (all higher expression in infants)

Supplementary Table 3. List of transcripts included in GSEA of Mtb-stimulated infant/adult AMϕ that have previously shown to be upregulated in Mtb-stimulated monocyte-derived macrophages from patients who had previously recovered from TB meningitis and pulmonary TB, relative to equivalents from patients with latent TB (2)

CCL1
CCL20
CCR2
*CXCL5**
*EREG**
*HAS1**
IL12B
*IL23A**
INHBA
*MMP1**
*PTGS2**
TNIP3

*Genes that were SDE between Mtb-stimulated infant vs adult AMϕs (all higher expression in infants)

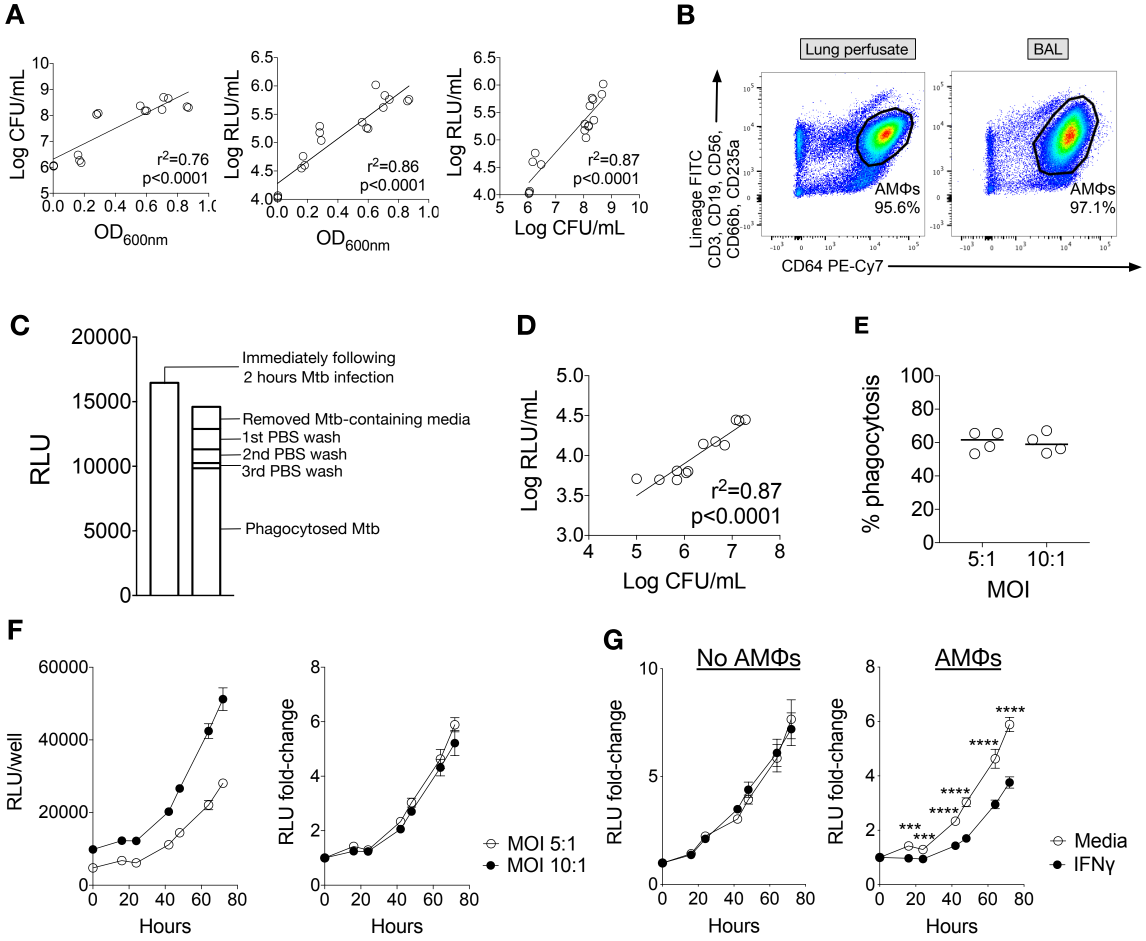

Supplementary Figure 1. Autoluminescent reporter *M. tuberculosis* facilitates the accurate measurement of phagocytosis and capacity to restrict mycobacterial replication by human AMϕs. (A) Correlation of CFU/mL and optical density (OD) with autoluminescence (RLU, relative light units) of Mtb-LuxG13 in liquid 7H9 culture; **(B)** Representative flow cytometry showing purity of AMϕs after 1-hour adherence of ex-vivo BAL cells (left) and BAL (right) cells followed by three PBS washes. **(C)** Autoluminescence of ex-vivo BAL AMϕs measured immediately following a 2-hour incubation with Mtb-LuxG13 at MOI 5:1 (left histogram), compared with a stack of measurements (right histogram) comprising of: the media removed from the same wells after the 2-hour incubation, three serial PBS washes and the remaining autoluminescence (representing phagocytosed intracellular bacilli). Data represents median of three replicate wells from a single donor. **(D)** Correlation of intracellular bacillary load and autoluminescence of ex-vivo BAL AMϕs from a single donor after 2-hour infection with Mtb-LuxG13 at MOI 5:1; **(E)** Comparison of proportion of phagocytosed Mtb-LuxG13 at MOI 5:1 and 10:1 in four replicate wells of ex-vivo BAL AMϕ from a single donor; **(F)** Comparative kinetics of mycobacterial replication of ex-vivo BAL AMϕs infected with MOI 10:1 or 5:1, expressed as serial RLU measurements over 72 hours (left graph shows absolute values, right graph shows fold change relative to zero time point); **(G)** Effect of IFNγ on mycobacterial replication, expressed as fold-change of serial RLU measurements of parallel wells of ex-vivo BAL AMϕ infected with Mtb-LuxG13 at MOI 5:1 (right graph) or bacillary inoculum alone without AMϕ (left graph), with/without recombinant human IFNγ 50ng/mL. Data points/bars represent median/range of RLU fold-change from four replicate wells relative to zero time point. Significance determined by 2-way ANOVA with correction for multiple comparison by Benjamini-Hochberg method.

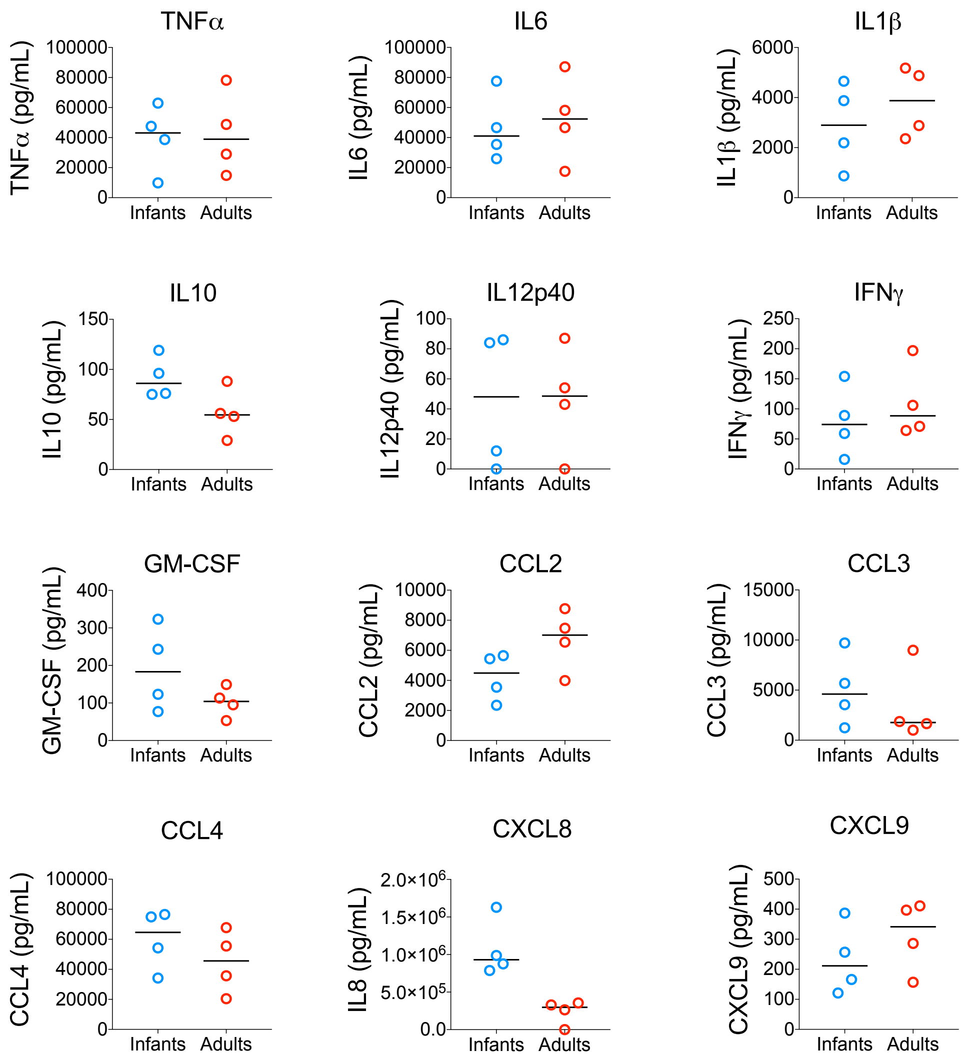

Supplementary Figure 2. Infant AMΦs exhibit dysregulated chemokine production. Soluble inflammatory mediator measurement by cytokine bead array of culture supernatants from Mtb H37Rv-stimulated infant vs adult AMϕs. Bar denotes median.

A

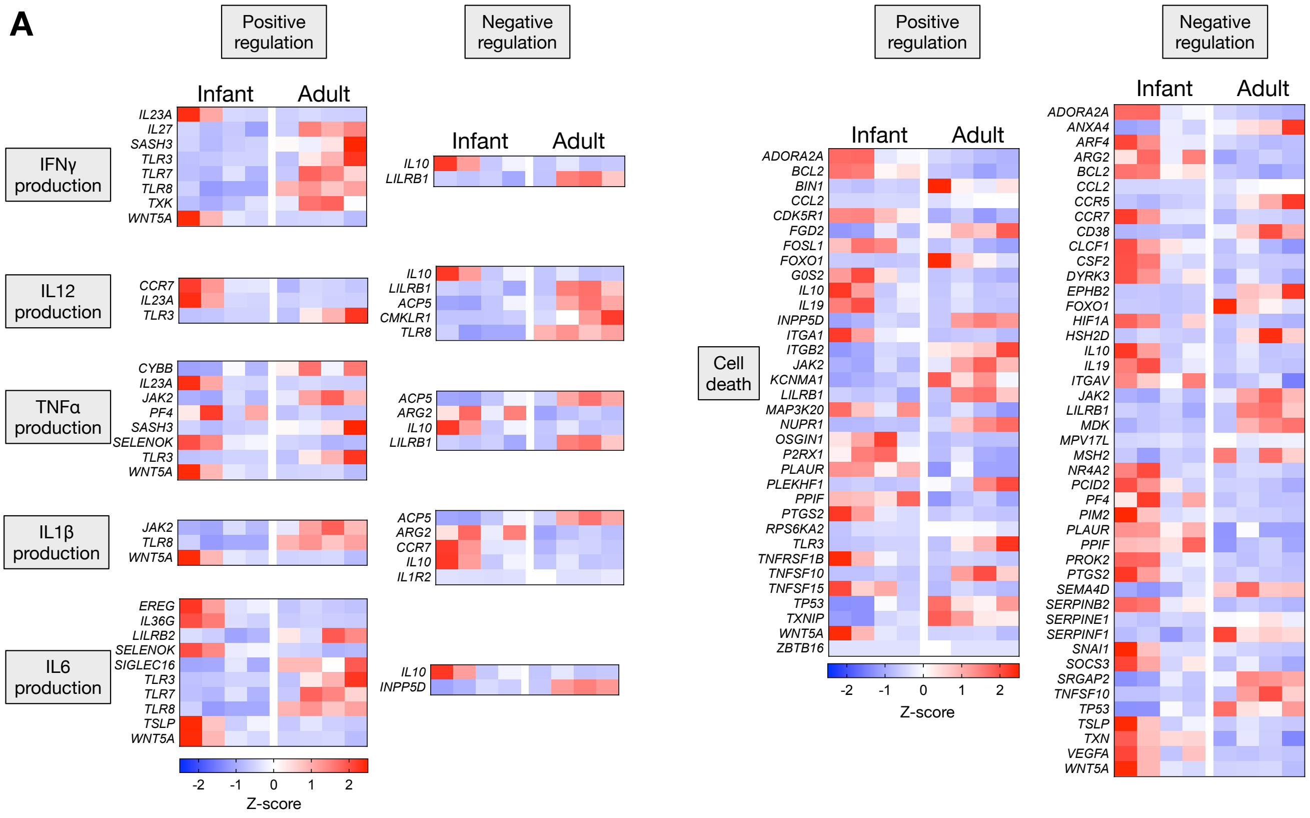

B

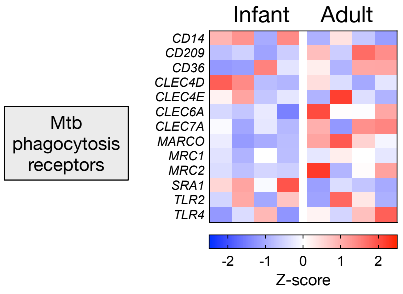

Supplementary Figure 3. Mtb-stimulated infant AMϕs do not exhibit polarized expression of genes involved in Mtb phagocytosis, innate cytokine production or cell death (A) Expression profile of SDE genes of Mtb-stimulated infant vs adult AMϕs involved in regulating production of IFNγ (GO:0032649), IL12 (GO:0032655), TNFα (GO:0032680), IL1β (GO:0032651) and IL6 (GO:0032675), and regulating cell death (GO:0010941). **(B)** Expression profile of SDE genes of Mtb-stimulated infant vs adult AMϕs encoding Mtb phagocytosis receptors. Scale intensity represents Z-score.

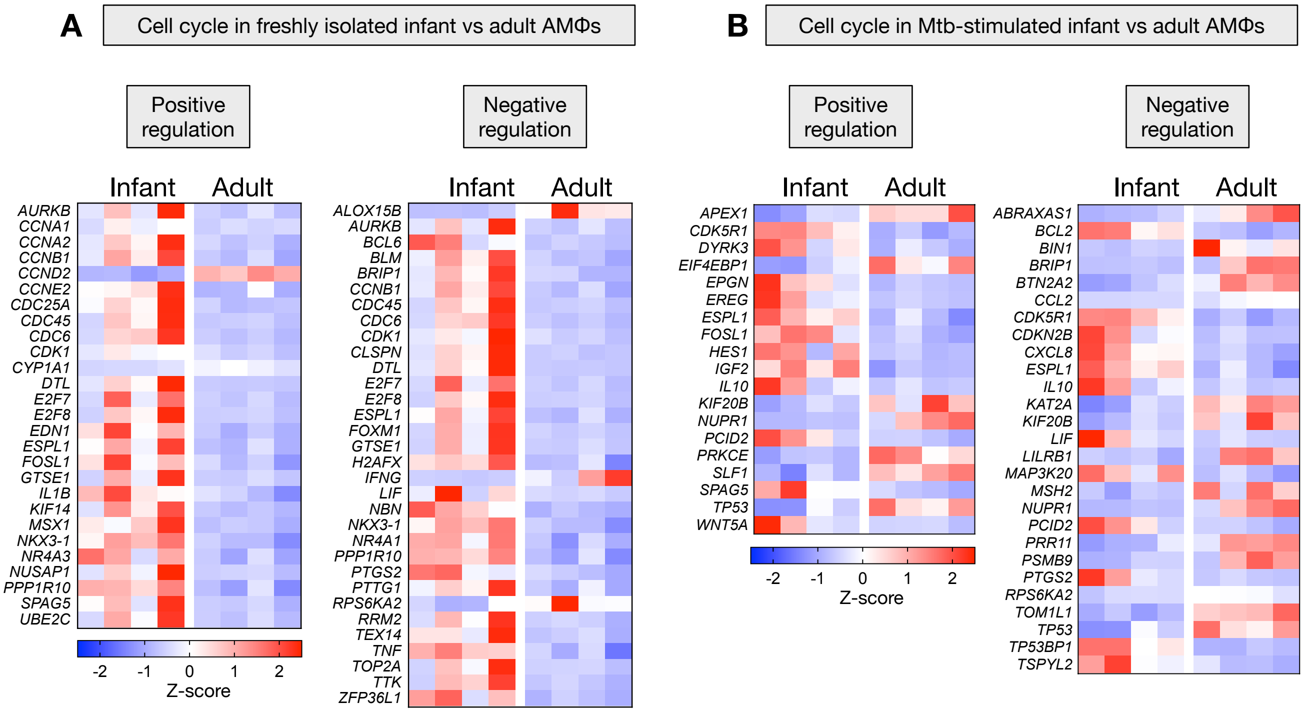

Supplementary Figure 4. Freshly isolated, but not Mtb-stimulated, infant AMϕs exhibit higher expression of genes involved in the regulation of cell cycle. Expression profile of SDE genes of infant vs adult AMϕs involved in the regulation of cell cycle (GO: 0045787 and 0045786) from (**A**) freshly isolated cells; and (**B**) Mtb-stimulated cells. Scale intensity represents Z-score.

Supplementary Video 1. Obtaining BAL during rigid bronchoscopy of an infant for investigation of suspected airway abnormality. The larynx was intubated under direct vision with a size 5Fr suction catheter. The suction catheter was advanced into the trachea alongside an endoscope to facilitate direct visualisation of endobronchial positioning. Sterile 0.9% sodium chloride solution was instilled into the suction catheter via a 16G intravenous catheter (JELCO, Smiths Medical). Following instillation, saline was immediately recovered into a sterile trap by applying gentle suction, and the procedure repeated on the opposite bronchus.

Supplementary References

1. Silver RF, Walrath J, Lee H, Jacobson BA, Horton H, Bowman MR, et al. Human alveolar macrophage gene responses to Mycobacterium tuberculosis strains H37Ra and H37Rv. *Am J Respir Cell Mol Biol* (2009) 40(4):491-504. Epub 2008/09/13. doi: 10.1165/rcmb.2008-0219OC. PubMed PMID: 18787177; PubMed Central PMCID: PMCPMC2660564.

2. Thuong NT, Dunstan SJ, Chau TT, Thorsson V, Simmons CP, Quyen NT, et al. Identification of tuberculosis susceptibility genes with human macrophage gene expression profiles. *PLoS Pathog* (2008) 4(12):e1000229. Epub 2008/12/06. doi: 10.1371/journal.ppat.1000229. PubMed PMID: 19057661; PubMed Central PMCID: PMCPMC2585058.
